# Phosphatidylcholine-specific B cells are enriched among atypical CD11c^high^ and CD21^low^ memory B cells in antiphospholipid syndrome

**DOI:** 10.3389/fimmu.2025.1585953

**Published:** 2025-06-03

**Authors:** Eduard Nitschke, Van Duc Dang, Hector Rincon-Arevalo, Franziska Szelinski, Jacob Ritter, Eva Schrezenmeier, Tobias Alexander, Tuan Anh Le, Yidan Chen, Annika Wiedemann, Jose-Bernardino Gonzalez, Andreia C. Lino, Ana-Luisa Stefanski, Thomas Dörner

**Affiliations:** ^1^ Department of Rheumatology and Clinical Immunology, Charité – Universitätsmedizin Berlin, corporate member of Freie Universität Berlin and Humboldt-Universität zu Berlin, Berlin, Germany; ^2^ German Rheumatism Research Centre, German Rheumatism Research Centre, Berlin, Germany; ^3^ Faculty of Biology, VNU University of Science, Vietnam National University, Hanoi, Vietnam; ^4^ Grupo de Inmunologiía Celular e Inmunogeneítica, Facultad de Medicina, Universidad de Antioquia UdeA, Instituto de Investigaciones Meídicas, Medelliín, Colombia; ^5^ Department of Nephrology and Medical Intensive Care, Charité – Universitätsmedizin Berlin, corporate member of Freie Universität Berlin and Humboldt-Universität zu Berlin, Berlin, Germany; ^6^ Berlin Institute of Health | Chariteí – Universitaütsmedizin Berlin, BIH Academy, Berlin, Germany; ^7^ Clinical Chemistry and Pathobiochemistry, Charité – Universitätsmedizin Berlin, corporate member of Freie Universität Berlin and Humboldt-Universität zu Berlin, Institute for Laboratory Medicine, Berlin, Germany; ^8^ Laboratoriumsmedizin & Toxikologie, Labor Berlin—Charité Vivantes GmbH, Berlin, Germany

**Keywords:** B cells, adaptive immunity, antigen-specific B cells, APS - antiphospholipid syndrome, anti-phospholipid antibodies, secondary APS, primary APS

## Abstract

**Background:**

Patients with antiphospholipid syndrome (APS) carry an increased risk of thrombosis and adverse pregnancy outcomes due to the presence of antiphospholipid autoantibodies (aPL). However, the pathogenesis of the disease remains incompletely understood regarding various aPL and the role of autoreactive B cells as precursors of antibody-secreting plasma cells (PC).

**Objective:**

To assess B-cell dysregulation in APS, with a focus on the distribution of B cell subsets and phosphatidylcholine (PtC)-specific cells.

**Methods:**

We used flow cytometry to study B cell subsets in peripheral blood mononuclear cells (PBMCs) from 20 healthy controls (HCs), 21 patients with primary APS (pAPS), and 16 patients with secondary APS (sAPS). A novel fluorescent liposome-based method was used to identify PtC-specific B cells in these subsets. Data were analyzed using manual gating and unsupervised clustering. We quantified aPtC antibody serum levels using ELISA and conducted correlation analyses between PtC-specific B cell subsets and aPL titers.

**Results:**

Patients with pAPS and sAPS exhibited significantly increased frequencies of atypical CD21^low^ and CD11c^high^ B cells, including PtC-specific B cells. Notably, both total and unswitched memory PtC-specific B cells were elevated in pAPS patients and correlated with aPL antibody titers. Unsupervised clustering further highlighted the increased frequencies of PtC-specific CD21^low^CD11c^high^ unswitched and switched memory B cells in both pAPS and sAPS.

**Conclusion:**

The enrichment of PtC-specific B cells among CD21^low^ and CD11c^high^ atypical memory subsets, along with their correlation with aPL serum levels, suggest a linkage between these atypical memory B cell subsets and autoantibody producing cells in APS.

## Introduction

Antiphospholipid syndrome (APS) is an autoimmune disorder characterized by autoantibodies against phospholipids (aPL) that lead to a procoagulant state in affected individuals. Clinical manifestations include a variety of arterial, venous or small-vessel thromboembolic events as well as adverse pregnancy outcomes (1). The disease can either occur as primary APS (pAPS) or associated/secondary APS (sAPS) which is commonly associated with another underlying autoimmune condition, most frequently systemic lupus erythematosus (SLE). In rare cases, patients can present with a catastrophic APS, a course of the disease with recurrent life-threatening multi-organ failure and high mortality caused by severe thrombotic complications that is difficult to manage. To establish a diagnosis, it is essential to meet both serological and clinical criteria. Patients must test positive for at least one of the three serological criteria (anti-beta2-glycoprotein 1 (aß2GP1) and/or anti-cardiolipin (aCL), either of IgG or IgM isotype, and/or lupus anticoagulant (LA)) at two different time points separated by at least 12 weeks. In addition, there must have been a preceding clinical manifestation, the clinical criterion (2). Besides these classical manifestations, non-criteria clinical features like thrombocytopenia, aPL associated nephropathy, heart valve lesions and livedo reticularis (1, 3) are associated with a higher disease burden in APS and the need of a more intense therapy (4), as recently included in the revised EULAR/ACR 2023 APS Classification Criteria (5).

Current therapeutic strategies include the treatment of thrombosis and its prevention by anticoagulation and/or antiplatelet therapies; however, there are no established selective treatment options targeting pathologic memory B- and plasma cells as the origin of aPL. Attempts to use B cell and plasma cell depleting therapies did not have lasting effects on aPL titers but could ameliorate refractory thrombotic and non-criteria manifestations (6–10). Thus, the main challenge is to better understand the underlying abnormalities of B cell differentiation that ultimately fuel the pool of autoreactive APS plasma cells. Overall, the B cell distribution in APS has not been extensively studied and results from existing studies often show considerable variation (11).

In this study, we aimed to characterize the general B cell distribution in APS patients compared to healthy controls, placing particular emphasis on a previously unknown population of phosphatidylcholine (PtC)-specific B cells. PtC, a phospholipid ubiquitously found in eukaryotes, is a major component of cell membranes, among other functions. Also, we were looking for the presence of atypical B cells that express low levels of CD21 or high levels of CD11c and that are associated with different autoimmune conditions (12–14). CD11c, together with CD18, forms complement receptor 4 (CR4) and is expressed mainly on monocytes, macrophages, dendritic cells and some B and T cells. CD21, also known as complement receptor 2 (CR2), is expressed on B cells and follicular dendritic cells. There is no literature regarding atypical B cells in APS. These cells might be precursors of aPL-producing cells that could serve as proxies for the antiphospholipid pool in patients with APS as well as targets for potential therapeutic interventions in this entity.

## Materials and methods

### Study subjects

21 patients with primary APS (pAPS) and 16 with associated/secondary APS (sAPS) were enrolled from the outpatient clinic of the Department of Rheumatology and Clinical Immunology at Charité – Universitätsmedizin Berlin. Twenty healthy probands (HCs) served as controls. All enrolled subjects were at least 18 years of age. Patients were classified as APS according to the revised Sapporo criteria (2). Additionally, on the date the blood sample was obtained, pAPS had to test positive for at least one serological criterion. Patients with sAPS were required to present with an underlying rheumatologic disease (for detailed donor information, see **Table 1** and **Supplementary Table 1**) in addition to an APS diagnosis. All participants provided written informed consent, according to the approval of the ethics committee of the Charité – Universitätsmedizin Berlin (EA1/009/17). Peripheral blood samples (EDTA anticoagulated and serum tubes, BD Diagnostics) were collected during regular outpatient visits. Clinical patient data were extracted from electronic medical records. As LA can test false positive under anticoagulant therapy, some values are missing for patients who were already under therapy at the time of enrolment. Some patients were also tested for anti-phosphatidylserine (aPhs) antibodies, non-criteria autoantibodies associated with APS.

**Table 1:** Study cohort.

Group	HC	pAPS	sAPS
	n = 20	n = 21	n = 16
**age** (median [IQR])	42.50 [27.50, 63.75]	49.00 [42.00, 60.00]	50.50 [39.50, 58.75]
sex			
w	10	13 (62%)	16 (100%)
m	10	8 (38%)	0 (0%)
underlying disease			
SLE	–	–	14 (88%)
SjD	–	–	1 (6%)
UCTD	–	–	1 (6%)
clinical manifestation			
venous only	–	4 (19%)	5 (31%)
arterial only	–	7 (33%)	3 (19%)
arterial + venous	–	9 (43%)	5 (31%)
undefined phenotype	–	1 (5%)	3 (19%)
pregnancy complication	–	4 (19%)	1 (6%)
aPL antibodies			
anti-β2GP1	–	18 (86%)	7 (44%)
anti-cardiolipin	–	19 (90%)	7 (44%)
lupus anticoagulant	–	16 (94%*)	4 (100%*)
*triple-positive*	–	*14 (67%)*	*3 (19%)*
medication			
low-dose acetylsalicylic acid	–	6 (29%)	2 (13%)
phenprocoumon	–	17 (81%)	7 (44%)
therapeutic heparin	–	2 (10%)	0 (0%)
antiplatelet agents/not ASS	–	1 (5%)	1 (6%)
glucocorticoids (1-20mg/d)	–	4 (19%)	12 (75%)
hydroxychloroquine	–	6 (29%)	9 (56%)
azathioprine	–	0 (0%)	5 (31%)
mycophenolate mofetil	–	0 (0%)	2 (13%)
methotrexate	–	0 (0%)	1 (6%)
tacrolimus	–	0 (0%)	1 (6%)
belimumab	–	0 (0%)	1 (6%)

*of all patients tested.

### Absolute numbers of B and T cells

Absolute numbers of B and T cells were measured in EDTA blood samples using 6-color TBNK Reagent (Becton Dickinson) and Trucount tubes (Becton Dickinson), according to the manufacturer’s recommendations. Samples were measured using a FACS Canto II flow cytometer (Becton Dickinson).

### PBMC isolation

Peripheral blood mononuclear cells (PBMCs) were isolated from EDTA blood by density-gradient centrifugation using Ficoll Paque Plus solution (GE Healthcare). EDTA blood (20 ml of EDTA blood were transferred to 35 ml with PBS, layered onto 15 ml of Ficoll Paque Plus, and centrifuged at 1800 rpm for 20 min. PBMCs were collected, washed with PBS, cell aggregates were removed, and cell counts were determined for further analyses.

### B cell and phosphatidylcholine – specific B cell staining

To identify PtC-specific B cells, we used 100 nm fluorescent liposomes, each labeled with one of two fluorochromes: Texas Red/CF-594 (FormuMax Scientific, Product code: F60103F-TR) or Oregon Green (OG) 488 (FormuMax Scientific, Product code: F60103F-OG). The liposomes are made of 1,2-dioleoyl-sn-glycero-3-phosphocholine (54mol), cholesterol (45 mol) and Oregon Green® 488 1,2-dihexadecanoyl-sn-glycero-3-phosphoethanolamine (1mol; Invitrogen O-12650). Freshly isolated PBMCs (2x10 (6)) were subjected to LIVE/DEAD staining dye BUV-395 (Thermo Fisher Scientific) for 30 min, followed by washing with PBS/BSA/EDTA. PBMCs were stained with a mixture of antibodies, including BUV711-CD19, BUV737-CD11c, BUV395-CD14, BUV395-CD3, BUV786-CD27, BUV-605-CD24, BUV510-CXCR5, BUV421-IgM, PE-Cy7-IgG, PE-CD21, APC-CD38, and APC-Cy7-IgD (all from Becton Dickinson). Simultaneously, 1 µL of PtC-CF594 (from a 1/80 dilution in PBS) and 1 µL of PtC-OG (from a 1/50 dilution in PBS) were added. After staining for 15 min at 4°C, the cell suspensions were washed once with PBS/BSA/EDTA and analyzed using a Fortessa X-20 Cytometer (Becton Dickinson). We accounted for inter-day variations in laser intensities using rainbow calibration beads (Thermo Fisher Scientific). Four sAPS had missing values for CD11c measurements owing to technical limitations on the day of analysis. More detailed information regarding the antibodies can be found in **Supplementary Table 3**.

### Gating strategy

The gating strategy is provided in **Supplementary Figure 1A**. For the analysis and classification of B cell subsets, we first gated lymphocytes based on their size (forward scatter, FSC) and granularity (sideward scatter, SSC). Doublets were excluded by both FSC and SSC. Live CD3^-^CD14^-^ lymphocytes expressing CD19 were considered B cells. CD27^high^CD38^high^ were classified as plasmablasts and within all non-plasmablasts, CD24^int^CD38^high^ B cells were classified as transitional B cells. Based on the expression of IgD and CD27, non-plasmablast, non-transitional B cells were subdivided into naive (IgD^+^CD27^-^), unswitched memory (IgD^+^CD27^+^), switched memory (IgD^-^CD27^+^), and double-negative (DN; IgD^-^CD27^-^) B cells.

### Blocking of PtC-specific binding sites

To identify B cells specific for PtC, we performed a competitive binding assay using unconjugated PtC liposomes. Briefly, isolated PBMCs were stained using LIVE/DEAD staining dye (Thermo Fisher Scientific). After 30 min, the cells were washed with PBS/BSA/EDTA, and non-fluorescent control liposomes (FormuMax Scientific, Product Code: F60103-P) were added to the pellet of PBMCs at increasing concentrations relative to the amount of fluorescent liposomes used for staining PtC-specific B cells. PBMCs were incubated for 30 min at 4°C. The samples were washed once with PBS, BSA, and EDTA. Staining of PtC-specific B cells was performed as described above.

### aPL titers and anti-PtC ELISA

The values for aß2GP1, aCL, aPhs, and LA were determined using ELISA (Orgentec Diagnostika GmbH) and diluted Russel’s Viper Venom Time Test (dRVVTT, Siemens Healthcare GmbH) as part of the routine laboratory. To investigate phosphatidylcholine-specific antibodies in the sera of subjects, we used commercially available ELISA for IgM, IgG (both Cusabio Technology LLC), and IgA (AFG Bioscience) isotypes. Serum samples were diluted, added to pre-coated ELISA plates together with controls, and incubated for 30 min at 37°C. The plates were washed five times with washing buffer. The HRP-conjugate was added, and the plates were incubated at 37°C for 30 min. The samples were washed five times, according to the manufacturer’s recommendations. Subsequently, substrates A and B were added and left to react for 10–15 min. at 37°C, depending on the isotype. The stop solution was added to each well and the optical density (OD) was immediately measured at 450 nm. Values greater than twice the standard deviation of the negative control were considered positive.

### Data analysis and statistics

Flow cytometry data were analyzed manually using the FlowJo software (Version 10, Becton Dickinson). Unsupervised identification of cell clusters based on flow cytometric data using the FlowSOM algorithm was performed using publicly available packages in R (version 4.3). For data preparation and running FlowSOM (15), the Spectre package and Seurat in R were used (16, 17). Prior to clustering, all CD19+ cells were gated in FlowJo and exported to.csv files containing the fluorescence values for each marker at single-cell level. In addition, PtC-specific B cells were prepared and exported accordingly. Fluorescence values were first transformed using Arcsinh transformation with a cofactor of 1000. FlowSOM clustering was performed using a concatenated file containing ~1.65 million B cells from all samples, based on surface markers IgD, IgM, CD24, CD27, CD38, CD21, and CD11c. This revealed nine clusters, two of which were merged with another metacluster, owing to minor differences in the expression of surface markers that did not allow for classification as a distinct cluster. Pregated PtC-specific B cells were identified within the dataset of all B cells, based on matching fluorescence values. Uniform Manifold Approximation and Projection (UMAP) coordinates were calculated based on the same surface markers used in FlowSOM clustering with a random subset of 120'000 B cells (40’000 B cells per disease group). To quantify specific B cell frequencies among the different metaclusters, the number of B cells per metacluster was calculated as the percentage of total B cells per sample.

Statistical testing and visualization were performed entirely in R using different publicly available packages such as tidyverse (18), rstatix, corrplot, viridis. For comparisons between two groups as well as for multigroup pairwise comparisons, we used a non-parametric non-paired test (Wilcoxon rank-sum test). P-values were adjusted using the Benjamini-Hochberg correction if the number of groups did not exceed three; otherwise, Bonferroni correction was applied. For comparisons between groups, HCs served as the reference group. P values were considered positive when p < 0.05 (p value mapping: * p<0.05, ** p<0.01, *** p<0.001, **** p<0.0001). Spearman’s rank correlation was used to calculate correlation coefficients, and correlations were considered significant at p<0.05.

## Results

### Cohort and patient characteristics

For the current study, we included 21 pAPS, 16 sAPS patients and 20 age and sex-matched HCs with regard to the pAPS cohort. Most patients with pAPS and sAPS were female, which is representative for these patient groups. Regarding a high-risk aPL profile, 14 pAPS and 3 sAPS patients tested positive for all serological criteria (triple-positive APS), and 16 pAPS and 4 sAPS patients tested positive for LA. Detailed information regarding the aPL titers can be found in **Supplementary Figure 3**. Among the patients with pAPS, the majority experienced arterial thromboembolic events (n=7) or a combination of arterial and venous (n=9) events, while four patients had only venous thromboembolism. In the sAPS group, three patients had arterial, five had combined arterial and venous, and five had only venous thromboembolic events. Four pAPS and one sAPS patient reported a history of pregnancy complications related to APS. In addition to anticoagulation and/or antiplatelet therapy, six pAPS and nine sAPS patients received hydroxychloroquine. The patients with a sAPS received more often glucocorticoids (max 20 mg/day) and other immunosuppressants. More detailed information regarding the study participants and their medications are provided in **Table 1** and **Supplementary Table 1**.

### APS patients show increased frequencies of atypical CD21^low^ and CD11c^+^ B cell subsets

**Figure 1:**
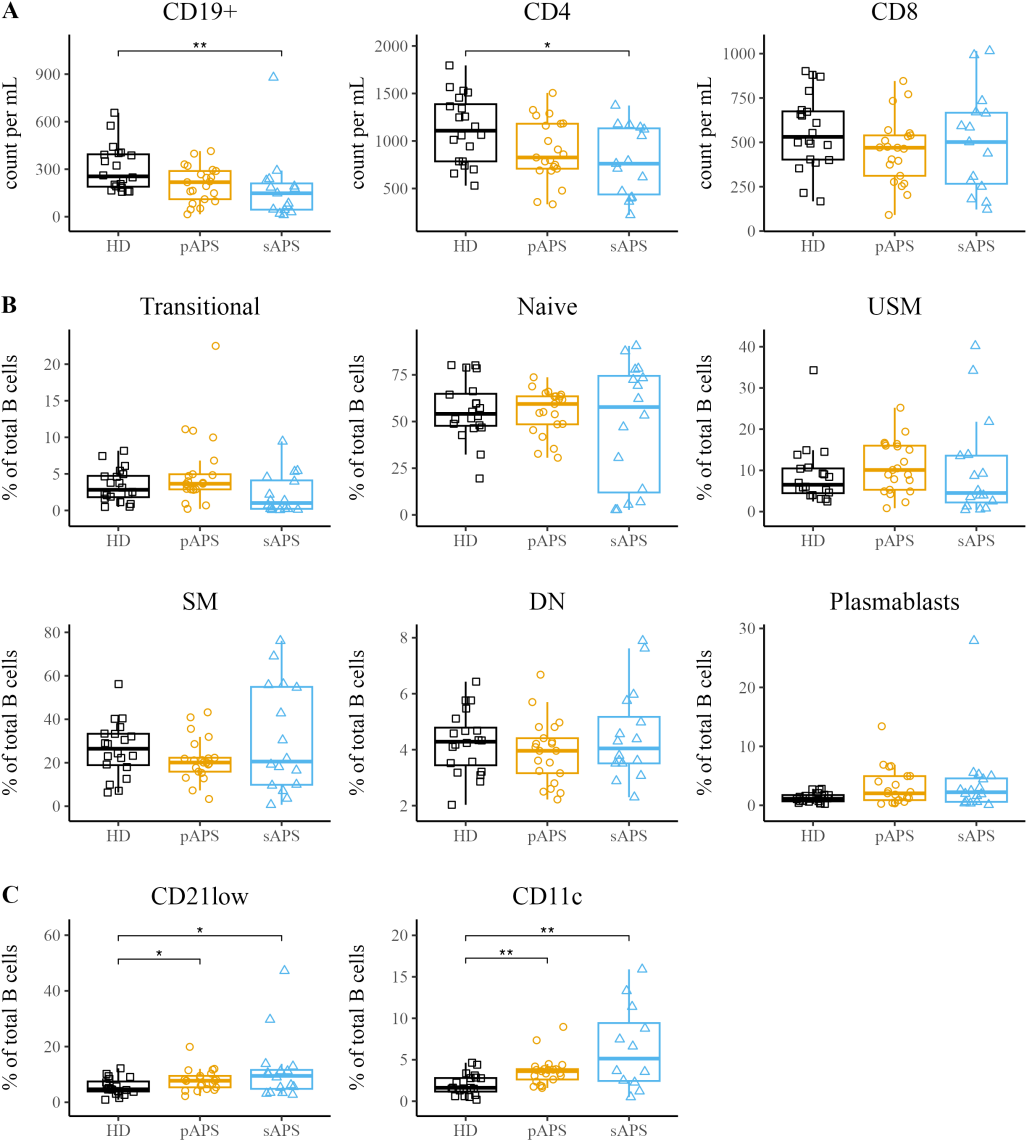
Increased atypical B Cell Frequencies in APS. **(A)** Assessment of absolute counts for CD19^+^ B cells and CD4^+^/CD8^+^ T cells in HCs, pAPS, and sAPS. **(B)** B cell subset distribution in APS: categorization according to surface marker expression into transitional (CD24^int^CD38^high^), naïve (IgD^+^CD27^-^), USM (IgD^+^CD27^+^), SM (IgD^-^CD27^+^), DN (IgD^-^CD27^-^) or plasmablasts (CD27^high^CD38^high^). A complete gating strategy is provided in **Supplementary Figure 1**. **(C)** Quantification of atypical CD21^low^ and CD11c^high^ B cells among the cohorts. APS, antiphospholipid syndrome; pAPS, primary APS; sAPS, secondary APS; HC, healthy control; USM, unswitched memory; SM, switched memory; DN, double negative. Statistics: Wilcoxon rank^-^sum test | reference group: HC | *p<0.05, **p<0.01.

First, we analyzed the total number of CD19^+^ B cells and CD4^+^ and CD8^+^ T cells among the groups (**Figure 1**). While total CD19^+^ B cells and CD4^+^ T cells were significantly decreased in patients with sAPS compared to those in HCs (CD19^+^: p<0.01, CD3+/CD4+: p<0.05, **Figure 1**), there were no differences between pAPS patients and HCs. Next, we performed flow cytometry stainings using 15 surface markers to investigate B cell subset distribution. B cell subsets were defined as transitional (CD24^int^CD38^high)^, naive (IgD^+^CD27^-^), unswitched memory (USM, IgD^+^CD27^+^), switched memory (SM, IgD^-^CD27^+^), so-called double negative (DN; IgD^-^CD27^-^) B cells, and plasmablasts (CD27^high^CD38^high^, gating strategy is shown in **Supplementary Figure 1**). In general, patients with pAPS and sAPS had a B cell subset distribution similar to that of HCs (**Figure 1**). Together with a variety of other B cell disturbances, increased frequencies of atypical B cells (CD11c^high^CD21^low^) have been reported in several chronic autoimmune diseases (12–14). Thus, we sought to investigate the presence of atypical memory B cells by the expression of surface molecules CD21 and CD11c. Interestingly, pAPS and sAPS patients showed significantly increased frequencies of CD21^low^ B cells compared to HCs (both p<0.05). The same applies to CD11c^high^ B cells, where we observed a significant increase among APS patients (both p<0.01, **Figure 1**).

### Detection of PtC-specific B cells

**Figure 2:**
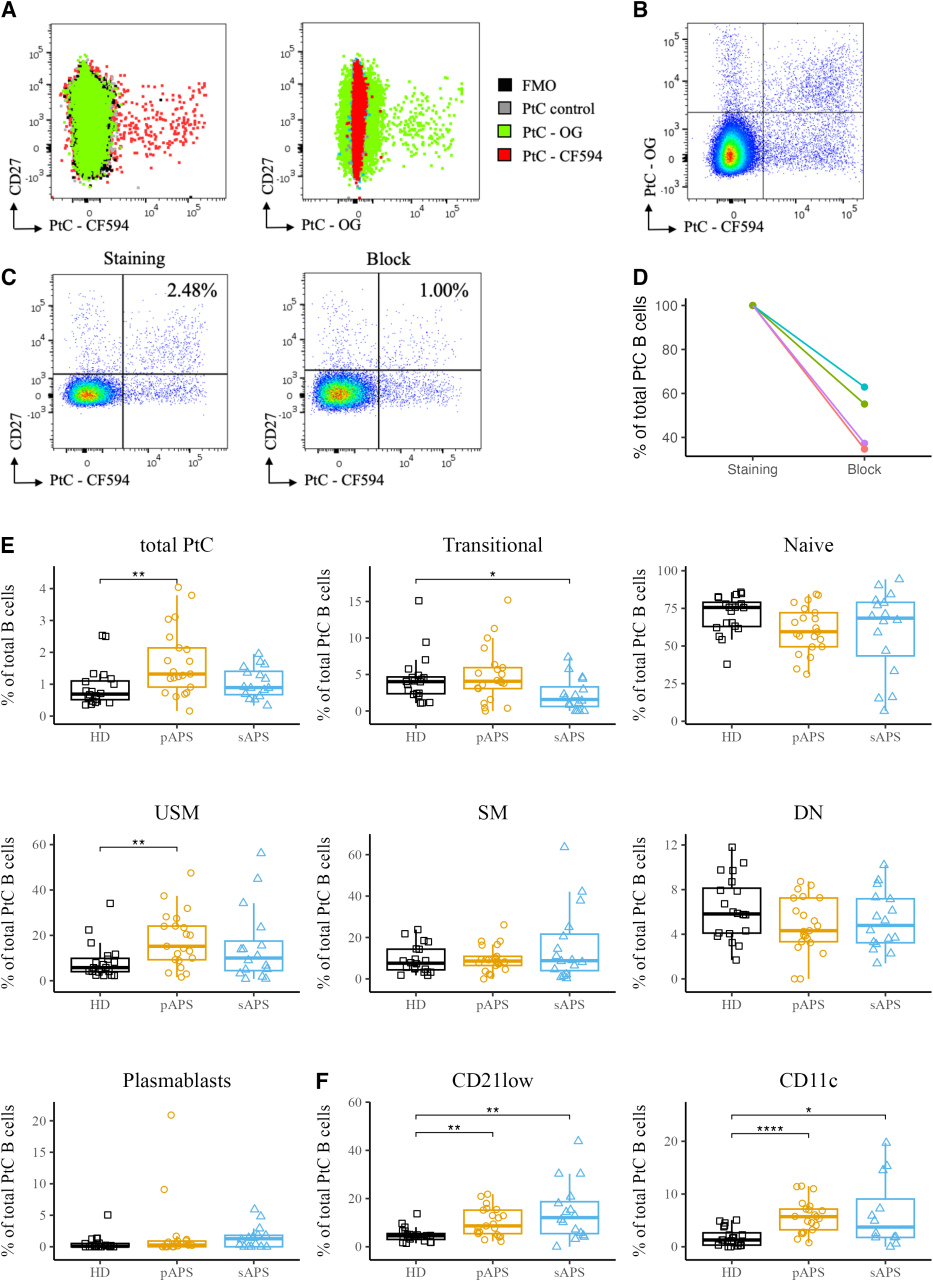
Specific staining and analysis of PtC-Specific B cells in pAPS using PtC liposomes. **(A)** Overlay of PtC-specific B cells stained with either CF594 labeled PtC liposomes, OG labeled liposomes, non-fluorescent control liposomes or without the addition of any liposomes (FMO). **(B)** Representative dot plot for the identification of PtC-specific B cells. Only B cells that are positive for both PtC-CF594 and PtC-OG were considered autoantigen-specific. **(C)** Two representative pseudocolor dot plots of PtC-specific B cells with a complete staining (left) and after blocking of specific binding-sites with non-fluorescent control liposomes (right). **(D)** Quantification of the relative decrease in PtC-specific B cells in 4 representative samples before- and after blocking with non-fluorescent control liposomes. **(E)** Frequencies of PtC-specific B cells and their distribution to major B cell subsets. B cell subsets were classified as in **Figure 1**. **(F)** Frequencies of atypical PtC^++^CD21^low^ and PtC^++^CD11c^high^ B cells among the cohorts. PtC, phosphatidylcholine; APS, antiphospholipid syndrome; pAPS, primary APS; sAPS, secondary APS; HC, healthy control; USM, unswitched memory; SM, switched memory; DN, double negative; OG, Oregon-Green. Statistics: Wilcoxon rank-sum testreference group: HC*p<0.05, **p<0.01, ****p<0.0001.

To investigate antigen-specific B cells in APS, we developed a flow cytometric method to identify B cells that recognize phosphatidylcholine (PtC), a prototypic phospholipid present in the cellular membranes. PtC liposomes labeled with Texas Red or Oregon Green were used in this study. Staining with non-fluorescent liposomes showed no signal in any of the relevant channels, and the fluorescent signals of both liposomes showed no interference (**Figure 2**). In addition, there was no signal with the FMO control, allowing for definite identification of the PtC-positive population (**Figure 2**). Double-positive cells were considered antigen-specific (**Figure 2**). To confirm the staining specificity, we conducted a blocking experiment with non-fluorescent liposomes using blood samples from four representative donors. For each subject, we observed a strong decrease in the frequency of PtC-specific B cells, with a mean decrease to 47.5%. (**Figure 2C**). For three representative samples we conducted a dose dependent block with increasing concentrations of blocking antigen that lead to a gradual decrease in the frequency of PtC-reactive B cells (**Supplementary Figure 1B**). To investigate whether the PtC containing liposomes randomly bind to the cell surface, we studied their binding to CD3+, CD14+ and DEAD cells, where we did not observe the binding pattern found for B cells (**Supplementary Figure 2**)

### Patients with pAPS show higher frequencies of PtC-specific B cells with a shift towards memory phenotype

Subsequently, we analyzed PtC-specific B cells among total B cells and major B cell subsets, as described above. Notably, pAPS patients showed significantly increased frequencies of PtC-specific B cells among total B cells compared to HCs (p<0.01, **Figure 2**), with a maximum of 4.04% of B cells being specific for PtC in a patient with pAPS. The majority of PtC-specific B cells carried a naïve phenotype across all groups, including controls. The frequency of PtC-specific transitional B cells was lower in the sAPS group than in HCs (p<0.05). We also found a significant increase in PtC-specific unswitched memory B cells in the pAPS group compared to HCs (p<0.01). Notably, PtC-specific B cells were significantly expanded among CD21^low^ and CD11c^high^ subpopulations in both pAPS and sAPS (**Figure 2**).

### PtC-specific B cells correlate with aPL titers

**Figure 3:**
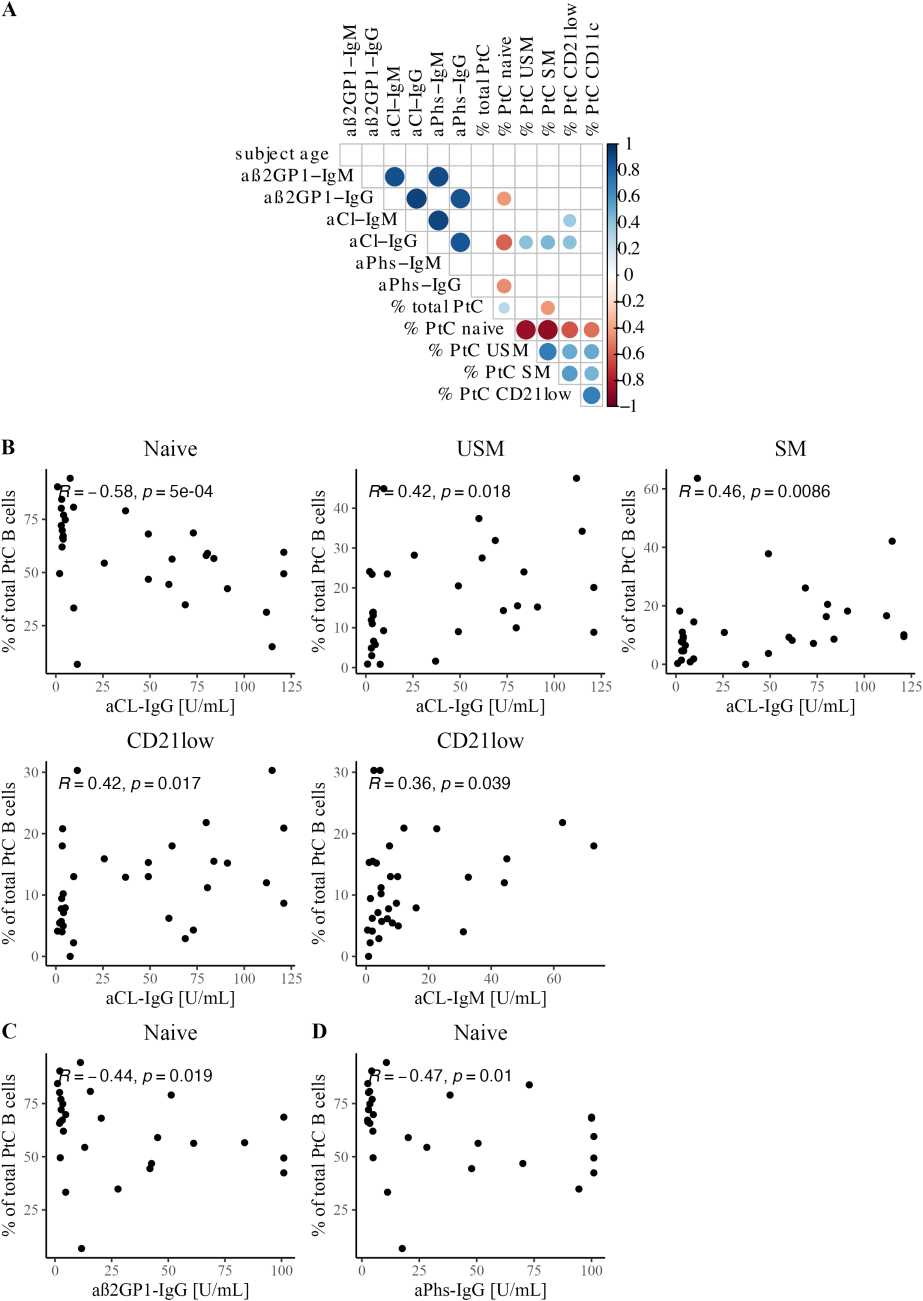
Correlation between PtC-specific B cells and serological features. **(A)** Correlation matrix shows Spearman correlation coefficients and significant correlations with p<0.05. **(B)** Scatterplots with Spearman correlation between aCL IgG and/or IgM titer and PtC-specific B cell subsets: naïve, USM, SM, and CD21^low^. **(C)** Scatterplot with Spearman correlation for aß2GP1-IgG and naïve PtC-specific B cells. **(D)** Scatterplot with Spearman correlation for aPhs-IgG and naïve PtC-specific B cells.

Next, we investigated the correlation between PtC-specific B cells and various serological markers. In particular, we analyzed the relationship between aPL serum levels and PtC-specific B cells in APS patients (**Figure 3**). Of note, this analysis revealed an inverse correlation between naive PtC-specific B cells and aCL-IgG (R = -0.58, p<0.0001, **Figure 3A**), aß2GP1-IgG (R = -0.44, p<0.05, **Figure 3A**) and aPhs-IgG (aPhs: R = -0.47, p <0.05, **Figure 3A**). In contrast, a positive correlation was found between unswitched and switched PtC-specific memory B cells and aCL-IgG serum levels (USM: R = 0.42, p<0.05; SM: 0.46 p<0.01 **Figure 3A**), as well as between atypic CD21^low^ PtC-specific B cells and aCL-IgG and aCL-IgM levels (aCL-IgM: R = 0.42, p<0.05; aCL-IgG: R = 0.36, p<0.05, **Figure 3A**).

These effects were confirmed by analyzing PtC-specific B cells in our APS cohort in relation to aPL positivity. PtC-specific naïve B cells were significantly diminished in patients who were positive for aß2GP1, aCL, or aPhs of either IgM and/or IgG (aß2GP1, p<0.01; aCL, p<0.0001; aPhs, p<0.001 **Supplementary Figures 4B, C**). At the same time, PtC-specific B cells were enriched among unswitched and switched memory compartments in patients positive for aCL or aPhs (aCL: both p<0.01; aPhs: USM: p<0.01, SM: p<0.05, **Supplementary Figures 4B, C**). The frequencies of CD21^low^ and CD11c^high^ PtC-specific B cells were increased in patients who tested positive for aCL antibodies (both p<0.05, **Supplementary Figure 4B**).

### Unsupervised clustering shows enrichment of PtC-specific B cells within memory B cells

**Figure 4:**
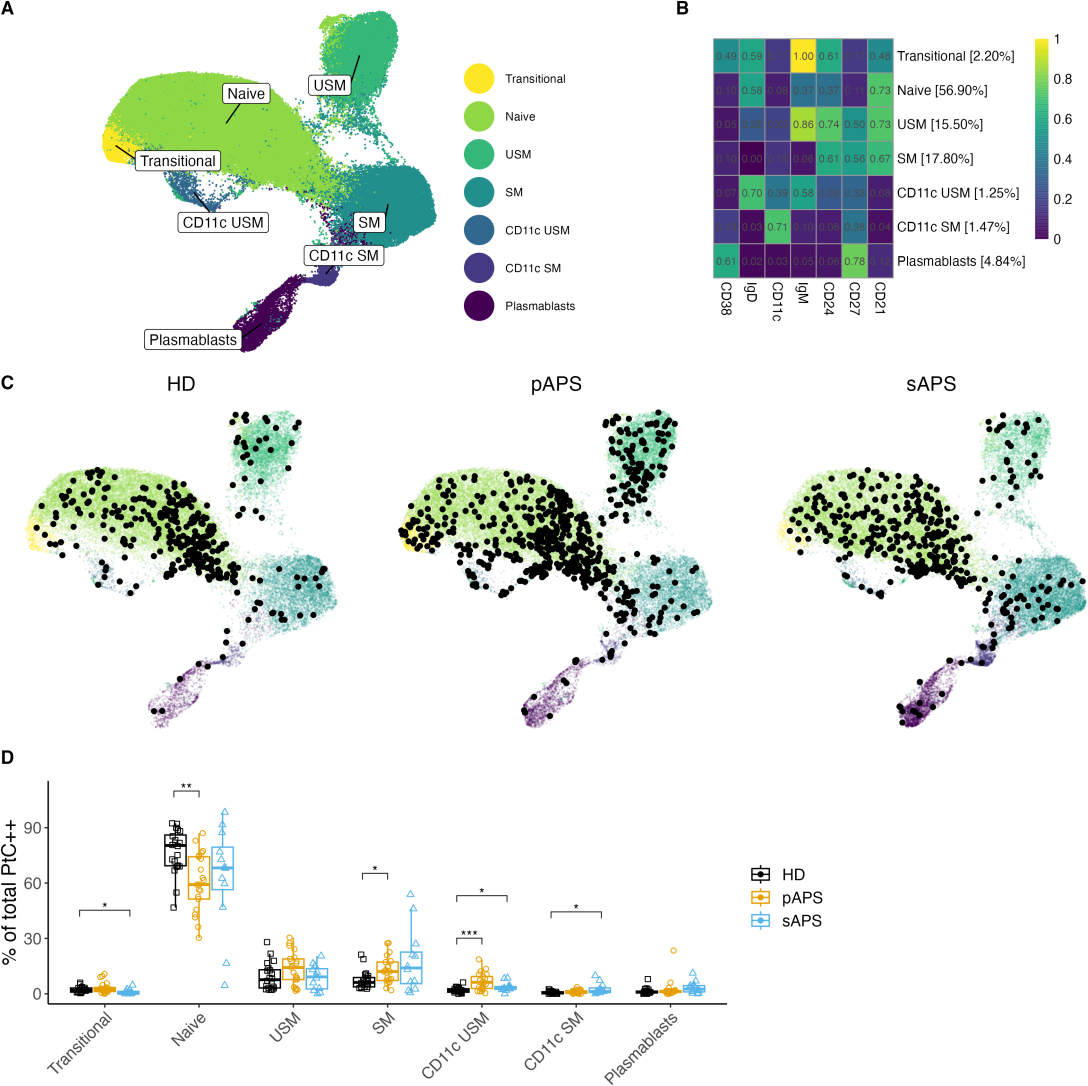
B cell clustering analysis in APS. **(A, B)** UMAP Visualization of B Cells: A representation of 120’000 B cells composed of a random subsample of 40’000 B cells from each group. Different colors represent B cell clusters identified using the FlowSOM algorithm. **(B)** Surface Marker Heatmap showing the expression of each surface marker within each FlowSOM metacluster. B cell clusters were classified according to certain expression profiles as follows: naïve, USM, SM, CD11c USM, CD11c SM, and plasmablasts. The percentages show the distribution of all B cells used for clustering to different clusters. **(C)** Individual UMAP graphs: Overlaid black dots represent all PtC-specific B cells within the random 40’000 per group subset. An increase in PtC-specific B cells in pAPS became evident both visually in the UMAP graphs and by cell count data in the subsample of B cells (PtC-specific B cell counts: HD: 347; pAPS: 613; sAPS: 392). **(D)** Distribution quantification: Analysis of the distribution of PtC-specific B cells to the FlowSOM metaclusters for each subject. UMAP, Uniform Manifold Approximation and Projection; PtC, phosphatidylcholine; PtC^++^, positive for both phosphatidylcholine liposome colors; APS, antiphospholipid syndrome; pAPS primary APS; sAPS, secondary APS; HC, healthy control; USM, unswitched memory; SM, switched memory; DN, double negative. Statistics: Wilcoxon rank sum test | reference group: HC | p-value adjustment: Bonferroni | *p<0.05, **p<0.01, ***p<0.001.

Next, we characterized the distribution of atypical CD21^low^ and CD11c^high^ PtC-specific B cells in the APS cohort. To overcome the limitations of cell numbers in manual analysis, we performed a multidimensional analysis using unsupervised clustering with all B cells and the FlowSOM algorithm in R based on the expression of surface markers. Seven distinct B cell clusters were identified (**Figure 4**). Clusters were characterized as follows: transitional, naive, unswitched memory, switched memory, CD11c unswitched memory, CD11c switched memory, and plasmablasts (**Figure 4A**, **Supplementary Figure 5A**). Interestingly, we were able to identify two CD11c^high^ clusters in the memory compartment (unswitched and switched memory), both distinguished also by CD21^low^ expression (**Figure 4**, **Supplementary Figure 5A**). In addition, there was an increased frequency of transitional B cells in pAPS as well as CD11c^high^ USM and SM subsets in sAPS **Supplementary Figure 5B**).

In the following step, we visualized the distribution of PtC-specific B cells across the seven clusters. Therefore, we calculated the UMAPs based on the fluorescence values of each clustering marker, thereby reproducing the FlowSOM metaclusters. The UMAP graphs by group showed interesting differences in the distribution of PtC-specific B cells across the seven clusters (**Figure 4**).

While PtC-specific B cells with a naïve phenotype were decreased (p<0.01), PtC-specific switched memory B cells were present at higher frequencies in pAPS (p<0.05, **Figure 4**). We also observed a significant increase in PtC-specific CD11c^high^ unswitched memory B cells in pAPS (p<0.001) and sAPS (p<0.05). Additionally, an increase in CD11c^high^ expressing switched memory B cells was observed in patients with sAPS (p<0.05, **Figure 4**).

### APS patients show increased serum levels of anti-PtC IgG and IgM antibodies

**Figure 5:**
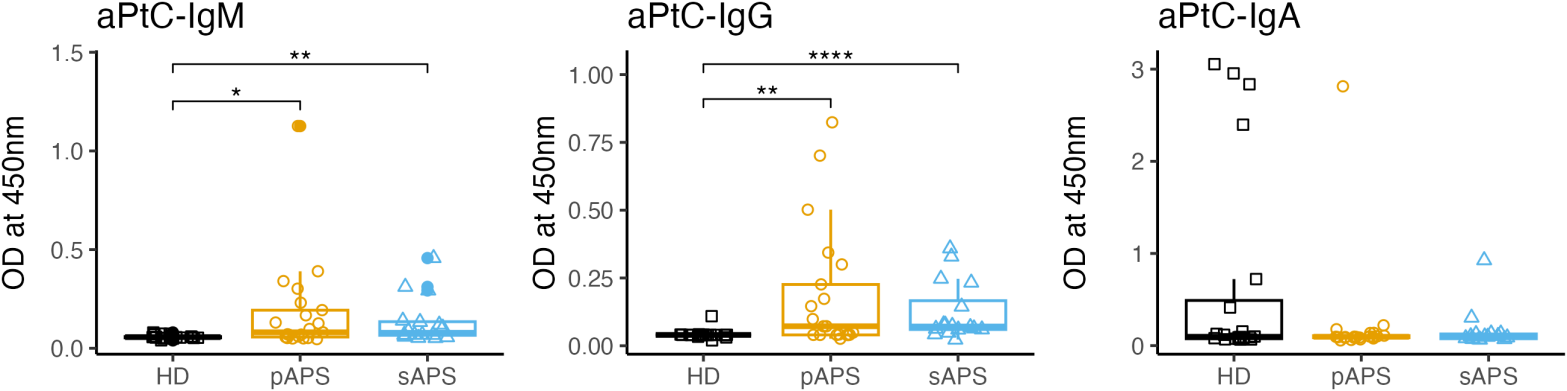
aPtC IgG, IgM and IgA levels among the groups. PtC, phosphatidylcholine; aPtC, anti-phosphatidylcholine antibodies; APS, antiphospholipid syndrome; pAPS, primary APS; sAPS. secondary APS; HC, healthy control; OD, optical density; ELISA, enzyme-linked immunosorbent assay. Statistics: Wilcoxon rank sum test | reference group: HC | p-value adjustment: Benjamini-Hochberg | *p<0.05, **p<0.01, ****p<0.0001.

To gain insight into the role of PtC-specific B cells in relation to the underlying autoreactive PC clones, specific anti-phosphatidylcholine antibody (aPtC) ELISAs were performed across the cohorts. Interestingly, in our cohort, aPtC-IgM and aPtC-IgG antibodies were only present in subjects with APS, who displayed significantly increased OD values for aPtC-IgM (pAPS: p<0.05; sAPS: p<0.01; **Figure 5**) and aPtC-IgG (pAPS: p<0.01, sAPS: p<0.0001, **Figure 5**). aPtC-IgA levels showed no significant differences among the three groups. Furthermore, in our cohort, some patients who were positive for aPtC antibodies also tested positive for conventional aPL (**Supplementary Table 1**).

## Discussion

Although APS is considered a B cell-mediated disease, and pathologic antiphospholipid antibodies are key pathogenic players, B cells and pathogenic PC clones, including their induction and maintenance mechanisms, remain poorly understood and studied in APS (11, 19, 20).

In this study, we investigated B cell subsets in patients with APS, focusing on B cells that specifically recognize PtC. Detection of PtC-specific B cells using PtC-containing fluorescent liposomes has been well established and utilized by us and multiple independent research groups (21–41). Notably, cloning and sequencing of VH genes of murine PtC-specific B cell receptors (BCRs) revealed that anti-PtC B cells predominantly utilize VH11 or VH12 genes, mostly belonging to the B1-cell lineage (42–44). In short, B1a cells in mice represent a B cell subset producing natural polyreactive autoantibodies that act as a first line defense in case of infection and are also known to play a pathogenic role in murine SLE (45, 46). By adapting a technique originally used in mice, we successfully established a method to identify human PtC-specific B cells, which were not characterized so far in APS patients.

By adapting this detection originally used in mice, we successfully established a method to identify human PtC-specific B cells, which were not characterized so far in APS patients. Interestingly, the presence of antigen-specific B cells reactive to PtC is not exclusive to patients with APS but can also be found in HCs. However, in pAPS, PtC-specific B cells carry a memory phenotype more frequently, while they are mainly naive in HCs, indicating dysregulated or impaired negative selection in patients with APS. We showed that atypical memory B cells expressing low levels of CD21 and high levels of CD11c were expanded in patients with APS, and PtC-specific B cells were enriched in these compartments. CD21^low^ B cells represent a heterogeneous group of atypical memory B cells that, in some scenarios, show a correlation with aging processes of the immune system, suggesting premature senescence in certain autoimmune conditions. CD21^low^ B cells and CD11c^high^ B cells are considered antigen-experienced atypical B cells that accumulate with age, during infections, and in chronic autoimmune diseases like SLE or Sjögren’s syndrome (47–51). This subset has gained significant attention due to the production of disease-specific autoantibodies in autoimmunity (12), often correlating with disease-specific manifestations, and in chronic infections they contribute to the production of disease-specific neutralizing antibodies (14, 52). Mechanistically, as suggested for SLE, these atypical B cells could expand at extrafollicular sites upon TLR7 ligation or under the influence of BAFF, IL21, and/or CD40 stimulation, but usually are not selected by follicular dendritic cells as in conventional germinal centers (53–55).

The presence of a small (0.5% of IgM^+^ B cells) pool of circulating aCL-specific B cells in healthy individuals was also postulated in a study on primary Ebstein-Barr virus (EBV)-infected healthy individuals and discussed as molecular mimicry or bystander activation of natural antibody-producing B cells (56). It seems that, in HC, peripheral tolerance mechanisms are able to prevent further differentiation of naive PtC-specific B cells into activated memory B cells and autoantibody-producing plasmablasts. The presence of autoreactive B cells of mostly naïve phenotype in HCs and their shift towards memory compartments in autoimmune diseases has also been shown in other studies on antigen-specific B cells: against thyroid peroxidase (TPO) in patients with Hashimoto thyroiditis or against citrullinated peptides (ACPA) in ACPA-positive RA (57, 58). APS patients seem to experience a loss of peripheral tolerance, promoting a shift of PtC-specific B cells into memory compartments, including CD21^low^ and CD11c^high^ atypical memory B cells. Our data suggest that this subset is also a reservoir of antigen-specific B cells and plasma cells in APS. Consistent with this finding, aPtC-IgG and aPtC-IgM antibodies were detected only in patients with APS.

Positive aPtC-IgA values were highest and most frequent in HCs, suggesting a mucosal origin. Interestingly, a molecular homology was recently found between ß2GP1 and the human gut commensal Roseburia intestinalis, suggesting mucosal crosstalk as a general promoter of cross-reactivity and the possible development of APS autoimmunity (59).

Only a few studies have been conducted on aPtC in humans, and their results are controversial. A recent study investigated different non criteria antibodies in APS and found that aPtC-IgG were associated with APS nephropathy (60). Another study investigated the role of aPtC in multiple sclerosis and found elevated levels of aPtC-IgM antibodies in active disease (esp. relapsing-remitting multiple sclerosis and clinically isolated syndrome) compared with clinically not active MS and controls (61). Another study investigated the presence of aPtC in healthy subjects and reported most subjects tested positive for aPtC-IgM, likely related to natural autoreactive B cells (62). However, these antibodies interacted with phosphorylcholine (not phosphatidylcholine), which may explain the difference to our results. The two molecules exhibit strong structural similarities, yet remain distinct entities. Other studies showed elevated aPtC antibodies in infectious diseases such as HIV and brucellosis, again supporting the conclusion that these reactivities may be part of natural defense mechanisms (63, 64). Beside different isotypes, further research is needed to better dissect which epitopes and/or posttranslational changes are related to these different functions. In summary, aPtC antibodies seem to have both regulatory and effector functions under healthy conditions, but an expanded pool of these autoreactive cells within atypical memory B cells could lead to pathogenic plasma cell induction in APS, where differential molecular characteristics compared to controls require further studies.

It is worth noting that lupus anticoagulant as well as anti-ß2GP1 antibodies are well-established as central players in APS pathogenesis, and their presence is of significant diagnostic and prognostic value (65, 66). In contrast, the role of non-criteria aPL, such as anti-PtC antibodies, remains less well-defined and continues to be an area of active investigation. Further research is needed to elucidate how these non-criteria antibodies might contribute to APS-related thrombotic events or other complications, either independently or in concert with the classic aPL.

Regarding differences between pAPS and sAPS patients in our cohort, two important messages must be acknowledged. On one hand, due to the underlying systemic autoimmune disease, there is a more pronounced general immune activation in sAPS than in pAPS which may include more pronounced alterations of the overall B cell subsets. On the other hand, immunosuppressive therapy is frequently used in patients with sAPS and can also influence the B cell subsets and our study results.

While providing valuable insights into antigen-specific B cell compartments in APS, some limitations must be acknowledged. First, the sample size was limited in this study. In addition, we could not measure all aPL specificities for each study participant, which may have influenced the serological data. To further validate the staining specificity, BCR cloning and detection of specific antibody secretion (e.g. using ELIspot assays) may be required to ensure the specificity of the PtC binding to the BCR. However, the current set of data follows earlier studies with different antigens where the underlying detection method has been successfully applied (67–70).

APS presents with a wide range of clinical manifestations. This study mainly focused on thrombotic APS. Future studies should investigate PtC-specific B cells in pure obstetric APS and non-criteria APS too. Also, it would be interesting to see how PtC-specific B cells occur in other disease constellations e.g. in patients with systemic lupus erythematosus without APS, aPL-negative thrombosis or even patients with APS that became seronegative over time. As the goal of this study was the first immunophenotypic characterization of PtC-specific B cells, future studies should address the functional characteristics of these cells, as well as their potential molecular and secretory capacities, including PtC-specific autoantibodies. Despite these limitations, this study provides a valuable basis for a better understanding of autoreactive plasma cells in APS and may guide future treatment principles.

In conclusion, our study revealed that patients with pAPS and sAPS exhibited higher frequencies of atypical CD21^low^ and CD11c^high^ expressing B cells than HCs. Notably, PtC-specific B cells have been reliably identified in humans by using fluorescent liposomes. These phospholipid-specific B cells were elevated in pAPS, exhibiting an atypical memory phenotype characterized by CD21^low^CD11c^high^ and correlated with aPL titers. Anti-PtC IgG and IgM antibodies were found in APS only. These results suggest that atypical PtC-specific B cells could be a characteristic autoreactive reservoir in APS, with potential for future therapeutic interventions.

## Data Availability

The raw data supporting the conclusions of this article will be made available by the authors, without undue reservation.

## Supplementary material

Supplementary Figure 1 Gating Strategy in FlowJo **(A)** Lymphocytes were gated based on their size and granularity using scattered light (FSC and SSC), and doublets were excluded. Cells expressing CD3 and/or CD14. Dead/unviable cells were excluded from analysis. All CD19^+^ cells were gated, and after exclusion of artifacts, they were classified as clean total CD19^+^ cells. Within the total B cells, CD21^low^ and CD11c^high^ B cells were gated, as well as PtC-specific B cells. Plasmablasts were characterized as CD38^high^CD27^high^ CD19^+^ cells. Among all non-plasmablast cells, transitional B cells were gated as CD38^high^CD24^int^ cells. All non-transitional cells were subdivided into IgD^+^CD27^-^ naïve, IgD^+^CD27^+^ USM, IgD^-^CD27^+^ SM, and IgD^-^CD27^-^ DN B cells. The plot titles represent the parent population and the names in the plot highlight the gated population. **(B)** Concentration-dependent blocking of PtC-specific binding sites on B cells. Increasing concentrations of non-fluorescent PtC-placebo liposomes have been added prior to the staining. The numbers correspond to the following amounts of PtC-control liposomes: Staining 0 nmol, 1 = 0.6875 nmol, 2 = 0.34 nmol, 3 = 6.875 nmol, 4 = 13.75 nmol, 5 = 137,5 nmol, Block = 275 nmol. PtC, phosphatidylcholine; USM, unswitched memory; SM, switched memory; DN, double negative; TR, Texas Red; OG, Oregon Green.

Supplementary Figure 2 Gating Strategy in FlowJo Frequency of PtC-liposome binding cells among CD3+, CD14+ and dead cells (bottom left) and CD19 positive cells (bottom right). The graph on top shows the matching parent gates. PtC, phosphatidylcholine; USM, unswitched memory; SM, switched memory; DN, double negative; TR, Texas Red; OG, Oregon Green.

Supplementary Figure 3 aPL Serum Levels in pAPS and sAPS Patients **(A)** aß2GP1 IgM and IgG levels in patients with pAPS and sAPS. **(B)** aCL IgM and IgG serum levels in pAPS and sAPS patients. **(C)** aPhs antibodies: aPhs IgM and IgG serum levels in patients with pAPS and sAPS. **(D)** Values for lupus anticoagulant in each group. Missing values occur due to anticoagulant therapy. aß2GP1, beta-2-glycoprotein 1 antibody; aCL, cardiolipin antibody; aPhs, phosphatidylserine antibody; LA, lupus anticoagulant; MFI, mean fluorescence intensity; TR, Texas Red; OG, Oregon Green; U/mL, units per milliliter. Statistics: Wilcoxon rank sum test | p-value adjustment: Benjamini-Hochberg | *p<0.05, **p<0.01, ***p<0.001, ****p<0.0001

Supplementary Figure 4 Characterization of aPL antibodies and their relation to PtC-specific B Cell subsets Distribution of PtC-specific B cell subsets based on the presence of **(A)** aß2GP1-IgM and/or aß2GP1-IgG antibodies, **(B)** aCL-IgM and/or aCL-IgG antibodies, and **(C)** aPhs-IgM and/or aPhs-IgG antibodies and **(D)** LA. Owing to missing values for LA (patients on anticoagulant therapy), no comparison could be achieved. PtC, phoshatidylcholine; aß2GP1, beta-2-glycoprotein 1 antibody; aCL, cardiolipin antibody, aPhs; phosphatidylserine antibody; LA, lupus anticoagulant; NA, not available. Statistics: Wilcoxon rank sum test | p-value adjustment: Benjamini-Hochberg | *p<0.05, **p<0.01, ***p<0.001, ****p<0.0001

Supplementary Figure 5 Distribution of B cells across FlowSOM metaclusters **(A)** Expression mapping on UMAP: Color-coded UMAP graphs highlight the expression of each surface marker in different parts of the UMAP graph. Values were scaled between 0 and 1 for each marker. **(B)** B cell distribution to different FlowSOM metaclusters: Left panel: comprehensive view of B cell clusters. Right panel: Focus on values ranging from 0 to 15%. PtC, phosphatidylcholine; APS, antiphospholipid syndrome; pAPS, primary APS; sAPS, secondary APS; HC healthy control; USM, unswitched memory; SM, switched memory; DN, double negative. Statistics: Wilcoxon rank sum test | reference group: HC | p-value adjustment: Bonferroni | *p<0.05 **p<0.01 ***p<0.001 ****p<0.0001

Supplementary Table 1 Patient characteristics Detailed characterization of all patients investigated in this study.

Supplementary Table 2 aPtC and conventional aPL Comparison of aPtC IgM, IgG, or IgA autoantibodies with conventional aPL (IgM/IgG aß2GP1, IgM/IgG aCL, or LA) and triple-positive APS among patients with pAPS and sAPS. PtC, phosphatidylcholine; APS, antiphosphospholipid syndrome; pAPS, primary APS; sAPS, secondary APS; aPtC, anti-phosphatidylcholine; ß2GP1, anti-beta2-glycoprotein 1; aCL, anti-cardiolipin.

Supplementary Table 3 Antibody clones Isotypes and clones of antibodies used for the staining. k, kappa.
