# Supplementary figures and images for: Phosphatidylcholine-specific B cells are enriched among atypical CD11chigh and CD21low memory B cells in antiphospholipid syndrome
Source: Front Immunol. 2025 Jun 3;16:1585953. doi: 10.3389/fimmu.2025.1585953 (PMC12170621; doi:10.3389/fimmu.2025.1585953)

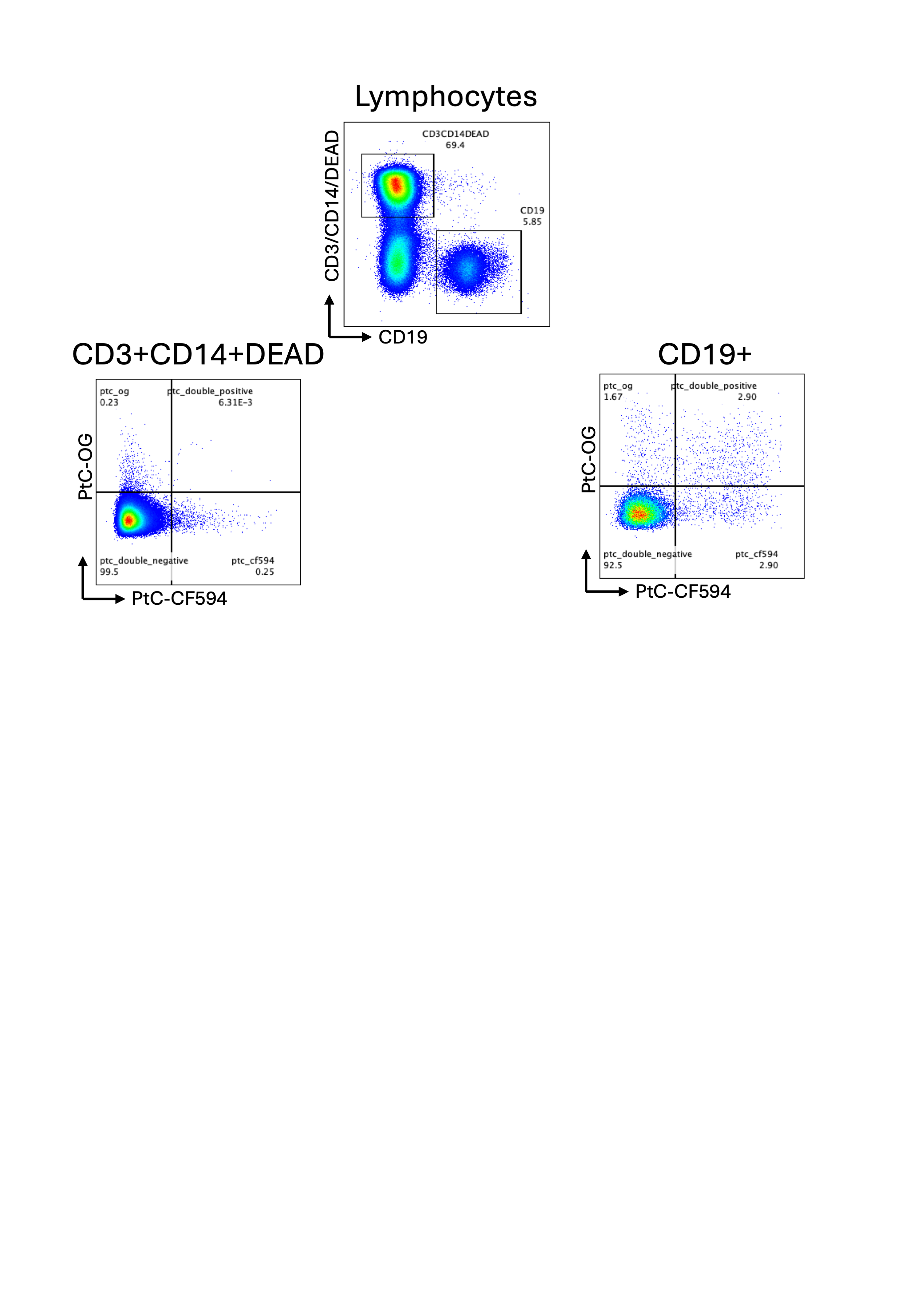

Supplement: Supplementary Figure 1 — Gating Strategy in FlowJo (A) Lymphocytes were gated based on their size and granularity using scattered light (FSC and SSC), and doublets were excluded. Cells expressing CD3 and/or CD14. Dead/unviable cells were excluded from analysis. All CD19+ cells were gated, and after exclusion of artifacts, they were classified as clean total CD19+ cells. Within the total B cells, CD21low and CD11chigh B cells were gated, as well as PtC-specific B cells. Plasmablasts were characterized as CD38highCD27high CD19+ cells. Among all non-plasmablast cells, transitional B cells were gated as CD38highCD24int cells. All non-transitional cells were subdivided into IgD+CD27- naïve, IgD+CD27+ USM, IgD-CD27+ SM, and IgD-CD27- DN B cells. The plot titles represent the parent population and the names in the plot highlight the gated population. (B) Concentration-dependent blocking of PtC-specific binding sites on B cells. Increasing concentrations of non-fluorescent PtC-placebo liposomes have been added prior to the staining. The numbers correspond to the following amounts of PtC-control liposomes: Staining 0 nmol, 1 = 0.6875 nmol, 2 = 0.34 nmol, 3 = 6.875 nmol, 4 = 13.75 nmol, 5 = 137,5 nmol, Block = 275 nmol. PtC, phosphatidylcholine; USM, unswitched memory; SM, switched memory; DN, double negative; TR, Texas Red; OG, Oregon Green. [file Image1.tiff]

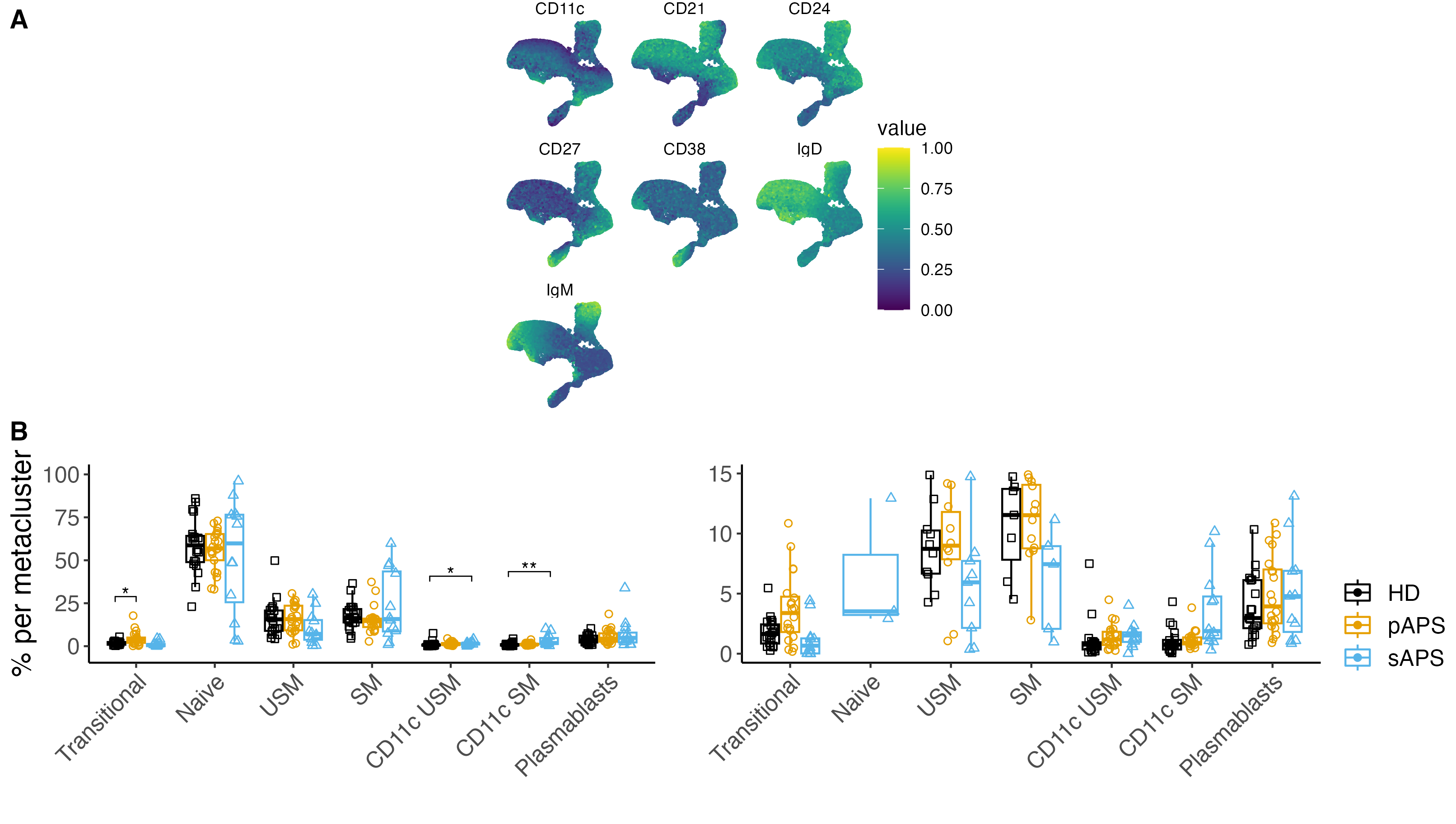

Supplement: Supplementary Figure 2 — Gating Strategy in FlowJo Frequency of PtC-liposome binding cells among CD3+, CD14+ and dead cells (bottom left) and CD19 positive cells (bottom right). The graph on top shows the matching parent gates. PtC, phosphatidylcholine; USM, unswitched memory; SM, switched memory; DN, double negative; TR, Texas Red; OG, Oregon Green. [file Image2.tiff]

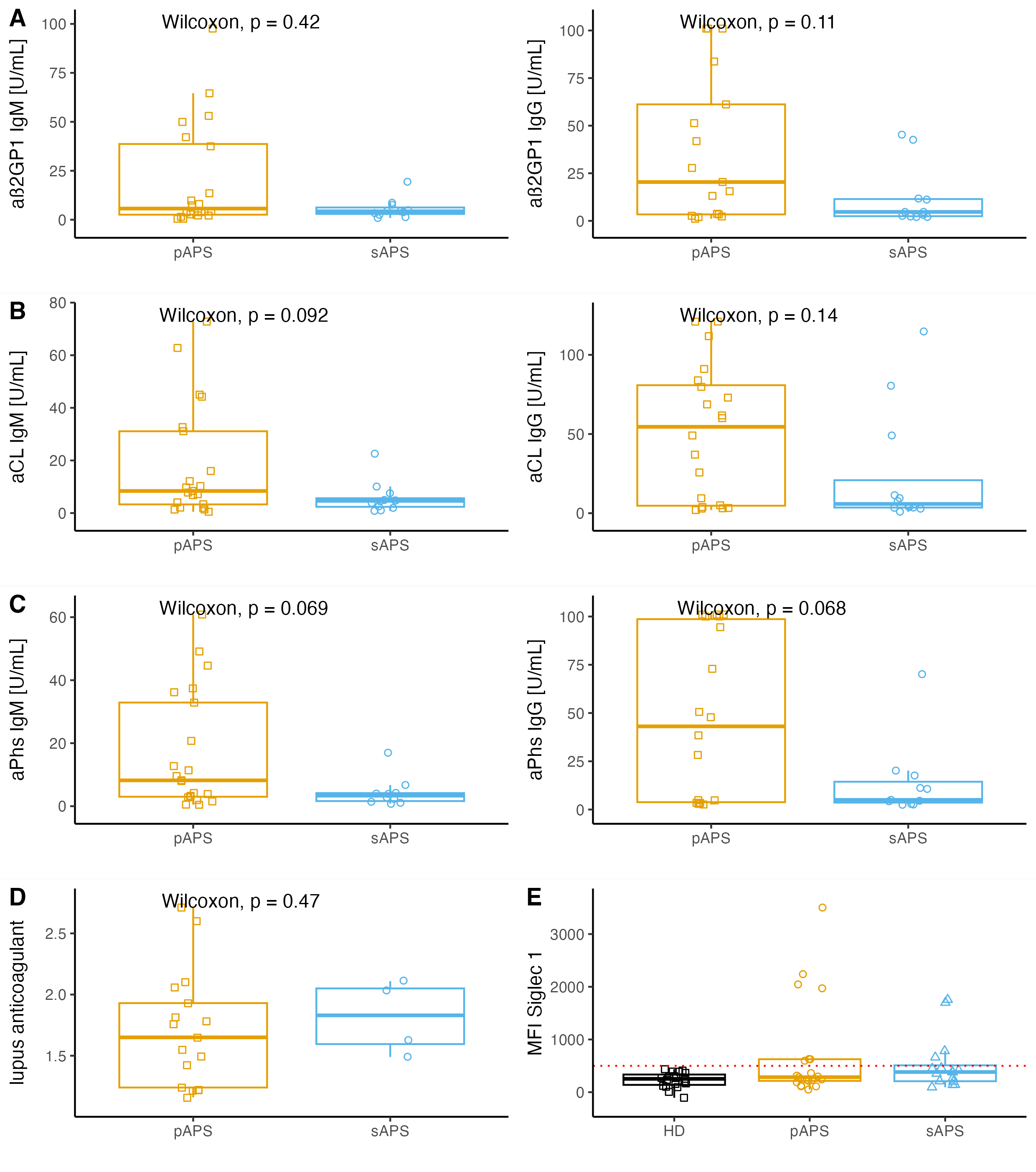

Supplement: Supplementary Figure 3 — aPL Serum Levels in pAPS and sAPS Patients (A) aß2GP1 IgM and IgG levels in patients with pAPS and sAPS. (B) aCL IgM and IgG serum levels in pAPS and sAPS patients. (C) aPhs antibodies: aPhs IgM and IgG serum levels in patients with pAPS and sAPS. (D) Values for lupus anticoagulant in each group. Missing values occur due to anticoagulant therapy. aß2GP1, beta-2-glycoprotein 1 antibody; aCL, cardiolipin antibody; aPhs, phosphatidylserine antibody; LA, lupus anticoagulant; MFI, mean fluorescence intensity; TR, Texas Red; OG, Oregon Green; U/mL, units per milliliter. Statistics: Wilcoxon rank sum test | p-value adjustment: Benjamini-Hochberg | *p<0.05, **p<0.01, ***p<0.001, ****p<0.0001 [file Image3.tiff]

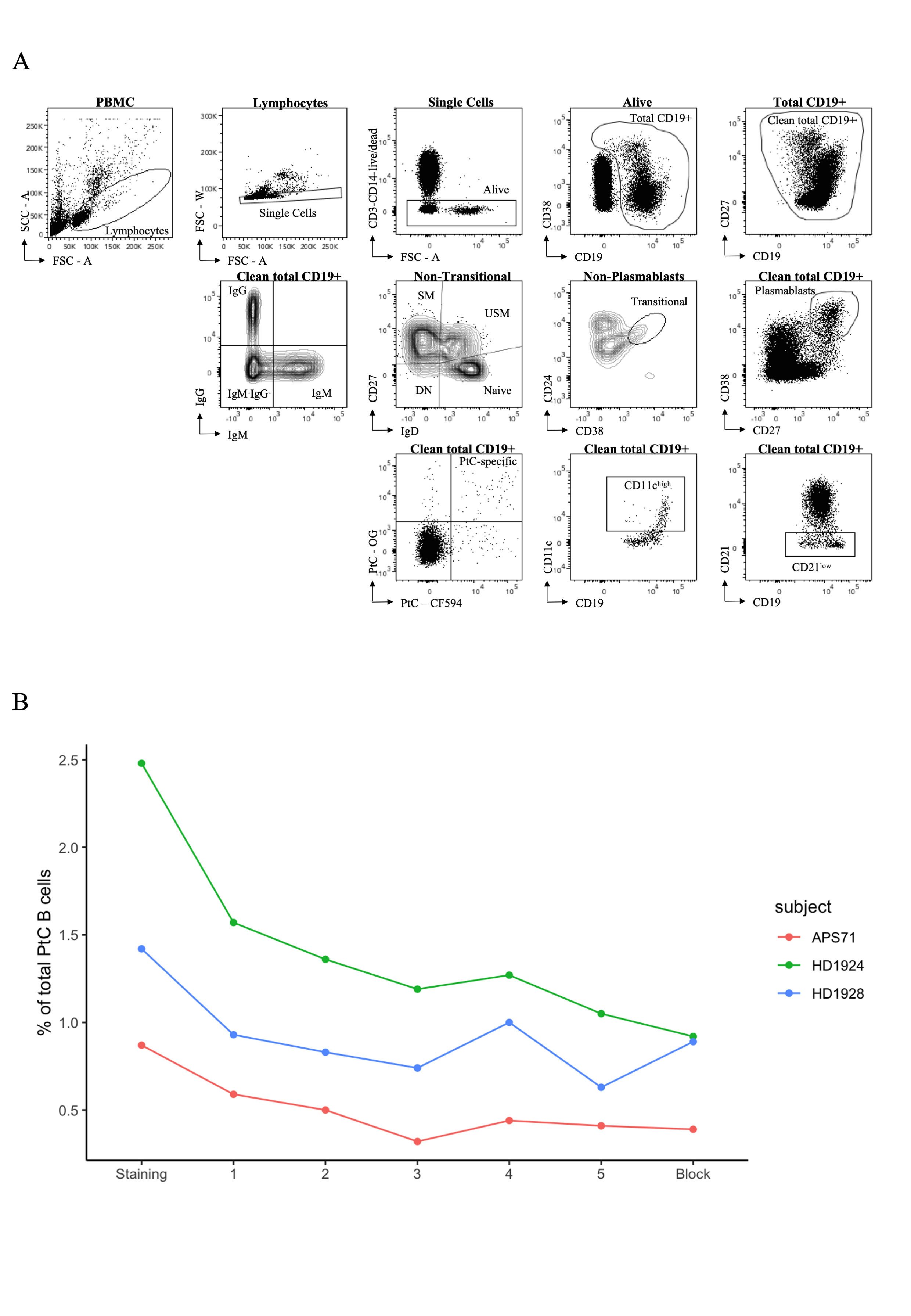

Supplement: Supplementary Figure 4 — Characterization of aPL antibodies and their relation to PtC-specific B Cell subsets Distribution of PtC-specific B cell subsets based on the presence of (A) aß2GP1-IgM and/or aß2GP1-IgG antibodies, (B) aCL-IgM and/or aCL-IgG antibodies, and (C) aPhs-IgM and/or aPhs-IgG antibodies and (D) LA. Owing to missing values for LA (patients on anticoagulant therapy), no comparison could be achieved. PtC, phoshatidylcholine; aß2GP1, beta-2-glycoprotein 1 antibody; aCL, cardiolipin antibody, aPhs; phosphatidylserine antibody; LA, lupus anticoagulant; NA, not available. Statistics: Wilcoxon rank sum test | p-value adjustment: Benjamini-Hochberg | *p<0.05, **p<0.01, ***p<0.001, ****p<0.0001 [file Image4.tiff]
