# Supplementary material for: Phosphatidylcholine-specific B cells are enriched among atypical CD11chigh and CD21low memory B cells in antiphospholipid syndrome
Source: Front Immunol. 2025 Jun 3;16:1585953. doi: 10.3389/fimmu.2025.1585953 (PMC12170621; doi:10.3389/fimmu.2025.1585953)
Supplement: Supplementary Table 1 — Patient characteristics Detailed characterization of all patients investigated in this study. [file Table1.docx]

| Supplement Table 1: Patient characteristics | | | | | | | | | |
| --- | --- | --- | --- | --- | --- | --- | --- | --- | --- |
| **ID** | **sex** | **thrombotic**  **manifestation** | | **preknown**  **obstetric manifestation** | **SLEDAI** | **medication** | **antibody category*** | |  |
| pAPS1 | w | arterial and  venous | no | | - | Phenprocoumon  Hydroxychloroquine | | 1 |  |
| pAPS2 | w | unknown | yes | | - | Hydroxychloroquine  Acetylsalicylic acid  Tinzaparin | | 2b  (LA missing) |  |
| pAPS3 | m | arterial | no | | - | Phenprocoumon | | 1 |  |
| pAPS4 | w | venous | no | | - | Phenprocoumon | | 1 |  |
| pAPS5 | m | arterial and  venous | no | | - | Phenprocoumon  Hydroxychloroquine | | 1 |  |
| pAPS6 | m | arterial and  venous | no | | - | Acetylsalicylic acid  Phenprocoumon | | 1 |  |
| pAPS7 | w | arterial and  venous | yes | | - | Phenprocoumon | | 1  (LA missing) |  |
| pAPS8 | m | arterial and  venous | no | | - | Phenprocoumon | | 1 |  |
| pAPS9 | w | venous | no | | - | Phenprocoumon | | 1 |  |
| pAPS10 | w | arterial and  venous | no | | - | Hydrochlorothiazide  Phenprocoumon  Budesonide | | 1 |  |
| pAPS11 | w | arterial and  venous | yes | | - | Acetylsalicylic acid  Phenprocoumon | | 1 |  |
| pAPS12 | m | venous | no | | - | Tinzaparin | | 1 |  |
| pAPS13 | w | arterial and  venous | no | | - | Phenprocoumon | | 1 |  |
| pAPS14 | w | venous | no | | - | Phenprocoumon | | 2b  (LA missing) |  |
| pAPS15 | w | arterial | no | | - | Ciclesonid  Metamizol  Phenprocoumon | | 1 |  |
| pAPS16 | w | arterial | no | | - | Hydroxychloroquine  Prednisolone  Phenprocoumon  Acetylsalicylic acid | | 1 |  |
| pAPS17 | m | arterial and  venous | no | | - | Phenprocoumon | | 2a |  |
| pAPS18 | m | arterial | no | | - | Clopidogrel  Acetylsalicylic acid  Prednisolone  Hydroxychloroquine | | 1  (LA missing) |  |
| pAPS19 | w | arterial | yes | | - | Acetylsalicylic acid  Mycophenolate mofetil  Phenprocoumon | | 1 |  |
| pAPS20 | w | arterial | no | | - | Phenprocoumon | | 1 |  |
| pAPS21 | m | arterial | no | | - | Phenprocoumon  Hydroxychloroquine | | 1 |  |
| sAPS1  (UCTD) | w | venous | no | | - | Phenprocoumon  Levothyroxin  Methotrexate  Prednisolone | | 1  (LA missing) |  |
| sAPS2  (SLE) | w | arterial | no | | ≤4 | Acetylsalicylic acid  Azathioprine  Prednisolone | | 1 |  |
| sAPS3  (SLE) | w | arterial | no | | ≤4 | Mycophenolate mofetil  Phenprocoumon  Clopidogrel  Prednisolone  Upadacitinib  Denosumab | | 1 |  |
| sAPS4  (SLE) | w | venous | yes | | 4 | Enoxaparin sodium  Hydroxychloroquine | | 2a |  |
| sAPS5  (SLE) | w | arterial and  venous | no | | ≤4 | Fondaparinux  Ibuprofen | | - |  |
| sAPS6  (SLE) | w | arterial and  venous | no | | ≤4 | Apixaban  Hydroxychloroquine  Acetylsalicylic acid | | - |  |
| sAPS7  (Sjögren) | w | arterial and  venous | no | | - | Prednisolone  Apixaban  Etoricoxib  Hydroxychloroquine | | - |  |
| sAPS8  (SLE) | w | venous | no | | ≤4 | Belimumab  Phenprocoumon  Prednisolone  Hydroxychloroquine | | 1  (LA missing) |  |
| sAPS9  (SLE) | w | arterial and  venous | no | | ≤5 | Eplerenone  Hydroxychloroquine | | - |  |
| sAPS10  (SLE) | w | venous | no | | 2 | Mycophenolate mofetil  Hydroxychloroquine  Prednisolone | | - |  |
| sAPS11  (SLE) | w | arterial | no | | 6 | Belimumab  Prednisolone  Hydroxychloroquine  Acetylsalicylic acid  Fluticasone/Salmeterol | | - |  |
| sAPS12  (SLE) | w | venous | no | | 2 | Hydroxychloroquine  Prednisolone  Azathioprine  Phenprocoumon | | - |  |
| sAPS13  (SLE) | w | unknown | no | | ≤4 | Phenprocoumon  Prednisolone | | 2c  (LA missing) |  |
| sAPS14  (SLE) | w | unknown | no | | 2 | Azathioprine  Prednisolone  Phenprocoumon  Hydroxychloroquine | | 2b  (LA missing) |  |
| sAPS15  (SLE) | w | arterial and  venous | no | | 4 | Azathioprine  Prednisolone  Nadroparin | | 1  (LA missing) |  |
| sAPS16  (SLE) | w | unknown | no | | ≤4 | Azathioprine  Prednisolone  Phenprocoumon | | 1 |  |

*Antibody category as advised by Miyakis, S., et al. (2006). J Thromb Haemost 4(2): 295-306.

*1: more than one laboratory*

*2a: LA alone*

*2b: aCL alone*

*2c: aß2GP1 alone*

LA *lupus anticoagulant*

UCTD *undifferentiated connective tissue disease*

unknown manifestation *thrombotic event has not been specified in the doctor’s letter*
