# Supplementary material for: Phosphatidylcholine-specific B cells are enriched among atypical CD11chigh and CD21low memory B cells in antiphospholipid syndrome
Source: Front Immunol. 2025 Jun 3;16:1585953. doi: 10.3389/fimmu.2025.1585953 (PMC12170621; doi:10.3389/fimmu.2025.1585953)
Supplement: Supplementary Table 2 — aPtC and conventional aPL Comparison of aPtC IgM, IgG, or IgA autoantibodies with conventional aPL (IgM/IgG aß2GP1, IgM/IgG aCL, or LA) and triple-positive APS among patients with pAPS and sAPS. PtC, phosphatidylcholine; APS, antiphosphospholipid syndrome; pAPS, primary APS; sAPS, secondary APS; aPtC, anti-phosphatidylcholine; ß2GP1, anti-beta2-glycoprotein 1; aCL, anti-cardiolipin. [file Table2.docx]

| Supplement Table 1: Conventional aPL and aPtC | | | | | | | | | | | | | | |
| --- | --- | --- | --- | --- | --- | --- | --- | --- | --- | --- | --- | --- | --- | --- |
|  | | aß2GP1 | | | aCL | | | LA | | | Triple positive | | |  |
|  |  | *pos* | *neg* | *NA* | *pos* | *neg* | *NA* | *pos* | *neg* | *NA* | *yes* | *no* | *NA* |  |
| aPtC-IgM | *pos* | 6 | 1 | 1 | 5 | 2 | 1 | 5 | 0 | 3 | 4 | 2 | 2 |  |
|  | *neg* | 19 | 7 | 3 | 21 | 5 | 3 | 15 | 1 | 13 | 13 | 9 | 7 |  |
| aPtC-IgG | *pos* | 7 | 0 | 3 | 7 | 0 | 3 | 6 | 0 | 4 | 6 | 0 | 4 |  |
|  | *neg* | 18 | 8 | 1 | 19 | 7 | 1 | 14 | 1 | 12 | 11 | 11 | 5 |  |
| aPtC-IgA | *pos* | 6 | 1 | 1 | 5 | 2 | 1 | 5 | 0 | 3 | 4 | 2 | 2 |  |
|  | *neg* | 19 | 7 | 3 | 21 | 5 | 3 | 15 | 1 | 13 | 13 | 9 | 7 |  |
