# Supplementary material for: Phosphatidylcholine-specific B cells are enriched among atypical CD11chigh and CD21low memory B cells in antiphospholipid syndrome
Source: Front Immunol. 2025 Jun 3;16:1585953. doi: 10.3389/fimmu.2025.1585953 (PMC12170621; doi:10.3389/fimmu.2025.1585953)
Supplement: Supplementary Table 3 — Antibody clones Isotypes and clones of antibodies used for the staining. k, kappa. [file Table3.docx]

| Supplement Table 3: Antibody clones | | |
| --- | --- | --- |
| **marker** | **isotype** | **clone** |
| CD19 | mouse IgG1k | SJ25C1 |
| CD27 | mouse IgG1k | L128 |
| CD38 | mouse IgG1k | HIT2 |
| CD24 | mouse IgG2ak | ML5 |
| CD21 | mouse IgG1k | B-ly4 |
| CD11c | mouse IgG1k | B-ly6 |
| IgD | mouse IgG2ak | IA6-2 |
| IgM | mouse IgG1k | G20-127 |
| IgG | mouse IgG1k | G18-145 |
| CXCR5 | rat IgG2bk | RF8B2 |
| CD3 | mouse IgG1k | UCHT1 |
| CD14 | mouse IgG2k | M5E2 |
